# Supplementary material for: In situ molecular weaving of ionic polymers into metal-organic frameworks for radioactive anion capture
Source: Nat Commun. 2025 Aug 11;16:7393. doi: 10.1038/s41467-025-62246-3 (PMC12339677; doi:10.1038/s41467-025-62246-3)
Supplement: Supplementary file 1 — Supplementary Information [file 41467_2025_62246_MOESM1_ESM.pdf]

Supplementary Information

In situ molecular weaving of ionic polymers into metal organic frameworks for radioactive anion capture

Xinghao Li [1], Xiang Lin [1], Zhenzhen Feng [1], Feng Chen [1], Qihang Huang[1], Linlin Zhen [1], Hongwei Wu [2], Jiayin Yuan [3], Yaozu Liao [1], and Weiyi Zhang [1]**

[1]State Key Laboratory of Advanced Fiber Materials, College of Materials Science and Engineering, Donghua University, Shanghai 201620, China

[2]College of Chemistry and Chemical Engineering, Donghua University, Shanghai 201620, China

[3] Materials Chemistry Division, Department of Chemistry, Stockholm University, Stockholm 10691, Sweden

*Corresponding authors: Yaozu Liao (yzliao@dhu.edu.cn)
Weiyi Zhang (wyzhang@dhu.edu.cn)

Experiment Section

1. Synthesis of MOFs

1.1 Synthesis of MOF_A(CuBTC)

Synthesis of MOF_A was performed according to the published method with slightly modified[1]. Typically, benzenetricarboxylic acid-H_3BTC (2.38 mmol, 0.5 g) was dissolved in 12 mL DMF-EtOH-water mixture (volume ratio = 1:1:1) to prepare Solution-A. Copper(II) chloride dihydrate (4.284 mmol, 0.7304 g) was dissolved in 12 mL of the same solvent to prepare Solution-B. Then, the Solution-B was added dropwise into Solution-A and stirred in room temperature for 15 min. After that, this mixture was heated up to 80 °C and stirred for 12 h to yield blue precipitate. The forming precipitate was collected by filtration, and then washed with DMF several times and subsequently with MeOH several times. The precipitate was then kept immersed in 30 mL MeOH and shaken for 24 h. That solvent was renewed three times by a new fresh solvent during this period of time. Finally, the samples were filtrated and dried at 60 °C under vacuum.

1.2 Synthesis of MOF_B(CuBDC)

Synthesis of MOF_B was performed according to the published method with slightly modified[2]. Typically, 1,4-benzenedicarboxylic-H_2BDC (1.51 mmol, 0.25 g) was dissolved in 15 mL DMF to prepare Solution-A. Copper(II) chloride dihydrate (1.812 mmol, 0.309 g) was dissolved in 10 mL water to prepare Solution-B. Then, the Solution-A was added dropwise into Solution-B and stirred in room temperature for 15 min. After that, this mixture was heated up to 80 °C and stirred for 12 h to yield blue precipitate. The forming precipitate was collected by filtration, and then washed with DMF several times and subsequently with MeOH several times. The precipitate was then kept immersed in 30 mL MeOH and shaken for 24 h. That solvent was renewed three times by a new fresh solvent during this period of time. Finally, the samples were filtrated and dried at 60 °C under vacuum.

1.3 Synthesis of MOF_C(CuTCPP)

Synthesis of MOF_C was performed according to the published method with slightly modified[3]. Typically, tetrakis (4-carboxyphenyl) porphyrin-TCPP (0.06068 mmol, 0.048 g) and copper(II) nitrate trihydrate (0.18204 mmol, 0.04398 g) are added into a mixture of 48 mL of DMF and ethanol (v/v = 3:1). After ultrasonication for 5 min, this mixture was heated up to 80 °C and stirred for 12 h to yield deep purple precipitate. The forming precipitate was collected by

filtration, and then washed with DMF several times and subsequently with EtOH several times. The precipitate was then kept immersed in 30 mL EtOH, and shaken for 24 h. That solvent was renewed three times by a new fresh solvent during this period of time. Finally, the samples were filtrated and dried at 60 °C under vacuum.

2. Sorption experiments

2.1 General procedures/equations of sorption

The standard Re stock solutions and competing-ion stock solutions were prepared by dissolving corresponding sodium salts in deionized water. The prepared Re stock solution and competing-ion stock solution were diluted to different concentrations for subsequent use. A typical batch sorption procedure was shown as follows and all batch experiments were conducted in a water-bathing with constant temperature vibrator at 25 °C. After shaking/stirring at a rate of 200 rpm for a desired period, the mixture was separated with a syringe filter unit (0.22 μm). The concentrations of residual ReO_4^- in the filtrate were analyzed by ICP-OES.

In the batch sorption experiments, the removal efficiency, RE (%) and sorption capacity at equilibrium, Q_e (mg g^{-1}) were calculated by the following equations:

$$RE = \frac{C_o - C_t}{C_o} \times 100\ \% \qquad (1)$$

$$Qe = \frac{C_o - C_e}{m} \times V \qquad (2)$$

Where C_0 (mg L^{-1}), C_e (mg L^{-1}) and C_t (mg L^{-1}) are the original concentration of ReO_4^-, adsorption equilibrium concentration of ReO_4^- and the concentration of ReO_4^- at a given time, respectively. V is the volume of solution (L), m is the mass of adsorbent (g).

2.2 ReO_4^- sorption data fitting by isotherm models

The sorption isotherm experiments of Ptriaz / MOF_C / MW-Ptriaz@MOF_C for ReO_4^- were carried out by mixing 5 mg of relevant adsorbents with 10 mL 100 ~ 2500 ppm initial ReO_4^- solution in a tube. The solution was shaken overnight to ensure the adsorption equilibrium. The samples were then filtrated and analyzed.

Two isotherm models, including Freundlich and Langmuir sorption models were used to fit the sorption data. As an empirical model, Freundlich adsorption model is based on the assumption that there are different binding energies between ReO_4^- and the heterogeneous surface sites of adsorbent. The adsorption isotherm model can be described mathematically as Equation (3).

$$Qe = k_F\, C_e^{1/n} \qquad (3)$$

Where C_e is the equilibrium concentration (mg L^{-1}), Q_e is the equilibrium adsorption capacity (mg/g), n is the Freundlich exponent, k_F (mg/g) is the Freundlich adsorption isotherm constant.

The Langmuir isotherm model is an ideal model with monolayer sorption on a homogeneous surface and adsorption sites of equal energy on the assumption. The adsorption isotherm model can be described mathematically as Equation (4).

$$\frac{C_e}{Q_e} = \frac{1}{Q_{max} \times k_L} + \frac{C_e}{Q_{max}} \qquad (4)$$

Where C_e is the equilibrium concentration (mg L^{-1}), Q_e is the equilibrium adsorption capacity (mg g-1), Q_{max} is the maximum adsorption amount at the time of equilibrium (mg g^{-1}), k_L (L mg^{-1}) is the Langmuir adsorption isotherm constant.

2.3 ReO_4^- sorption data fitting by kinetics models

The sorption kinetics experiments of MW-Ptriaz@MOF_C were conducted by collecting samples at different times. Typically, 50 mg of MW-Ptriaz@MOF_C was added to 50 mL of 25 ppm ReO_4^- aqueous solution in a beaker (m/V ratio is 1 g L^{-1}), followed by stirring at 200 rpm. Aliquots were taken from the mixture at appropriate time intervals ((1 min, 3 min, 5 min, 10 min, 15 min, 20 min, 30 min, 60 min, 90 min, 120 min, 150 min, 180 min, 360 min) and the sorbent was separated through a 0.22 μm membrane filter. The ReO_4^- concentration in the resulting solution was analyzed by ICP-OES. The Pseudo-first-order and Pseudo-second-order model were used to analyze sorption kinetics. The two models are expressed as followed.

Pseudo-first-order model:

$$ln(q_e - q_t) = lnq_e - k_1 t \qquad (5)$$

Pseudo-second-order model:

$$\frac{t}{q_t} = \frac{t}{q_e} + \frac{1}{k_2 q_e^2} \qquad (6)$$

where k_1 (min^{-1}) and k_2 (g mg^{-1} g^{-1}) are constants of pseudo-first-order model and pseudo-second-order model, respectively. q_t is the uptake amount of ReO_4^- at t min (mg g^{-1}), q_e is the uptake amount of ReO_4^- at equilibrium (mg g^{-1}), respectively.

2.4 Effect of pH

The effect of pH for ReO_4^- sorption of MW-Ptriaz@MOF$_C$ was evaluated by adding 5 mg of adsorbent to 10 mL of 25 ppm ReO_4^- solution at different pH (pH= 1, 3, 5, 7, 9, 11). The pH values were adjusted by NaOH (1M) or HNO_3 (1M) aqueous solutions. The samples were stirred for 6 h to ensure the equilibrium. And then the samples were filtrated and analyzed by ICP-OES.

2.5 Anion selectivity study

Effect of competing ions on the sorption properties of ReO_4^- was tested by adding different concentrations of $NaNO_3$, Na_2SO_4, NaCl, Na_2CO_3 and Na_3PO_4, respectively. The as-synthesized MW-Ptriaz@MOF$_C$ (10 mg) was added to 10 mL ReO_4^- solution (0.1 mM, m/V = 1 g L^{-1}) with concentrations of Cl^- (0.1 mM), concentrations of CO_3^{2-} (0.1 mM), concentrations of PO_4^{3-} (0.1 mM), varying concentrations of NO_3^- (0.1 mM, 1 mM, 10 mM, 50 mM) and varying concentrations of SO_4^{2-} (0.1 mM, 1 mM, 10 mM, 50 mM), respectively. After shaking at a rate of 200 rpm for 12 h, the mixture was filtrated for ICP-OES analysis.

2.6 Recyclability test

In the first cycle, 25 mg MW-Ptriaz@MOF$_C$ was added to in an aqueous solution of ReO_4^- (25 mL, 25 ppm). The solution was shaken for 6 h to ensure the adsorption equilibrium. After that, the concentration of ReO_4^- remaining in the water phase was determined by ICP-OES. Subsequently, the Re-loaded MW-Ptriaz@MOF$_C$ was immersed in a $NaNO_3$ solution (50 mL, 2 M) for 12 h. Then the regenerated adsorbent was filtered and put into the next run after washing by deionized water for three times and drying, respectively.

2.7 Stability test

Stability was studied by immersing MW-Ptriaz@MOF$_C$ into aqueous solution with different pH values (pH = 1, 3, 5, 7, 9, 11) for one day at ambient temperature. The solid was separated and dried for PXRD test.

2.8 Sorption in simulated Hanford low activity waste (LAW) system

A simulated Hanford LAW melter recycle solution was prepared according to reported methods[4], in which $^{99}TcO_4^-$ was replaced by ReO_4^-. In a batch experiment, MW-Ptriaz@MOF$_C$ was added into the simulated solution accompanied by different solid/liquid ratios. The mixture was shaken for 12 h and the concentration of ReO_4^- remaining in water phase was determined by ICP-OES.

3 Theoretical calculations

3.1 Molecular dynamics (MD) simulations of realignment of cation polymer chains

Firstly, cationic polymer chains (Ptriaz) comprising 48 head-tailed repeating units were initially established using Materials Studio. Subsequently, the amorphous cell module was utilized to model the coexistence of 4 cationic polymer chains. For the MOFs structure, we obtained the corresponding *cif* file from Chem Tube 3D and imported it into Materials Studio. Regarding the calculation parameters, this study employed the GAFF-UFF hybrid force field, wherein Cu was assigned the UFF atomic type while all other atoms are categorized under the GAFF atomic type. This choice of force field parameters has been extensively detailed in numerous studies concerning the adsorption and diffusion of organic molecules within MOFs. Atomic charges within the periodic structure of MOFs were computed using the CP2K program[5], employing DZVP-MOLOPT-SR-GTH as the basis group for Cu and TZVP-MOLOPTGTH for the remaining atoms. To maintain the system's overall charge neutrality, the REPEAT charge, known for its enhanced reproducibility in determining electrostatic potential, was employed. The MD simulations were conducted using GROMACS v.2019.6 software[6, 7]. Initially, the configuration was minimized employing the steepest descent method with a convergence criterion set at 500 kJ mol^{-1} $nm^{-}1$. Subsequently, 500 ps of simulations were executed on the NVT system at 298 K. Sampling of the system occurred at intervals of 0.1 ps, and equilibrium was determined based on variations in system energy, trajectory configuration analysis, with the last 100 ps of trajectories selected for result analysis. Temperature equilibration was achieved utilizing a V-rescale controller, gradually increasing the temperature from 0 to 298 K over the 100 ps preceding equilibrium to ensure a more plausible molecular conformation. Periodic boundary conditions are enforced along three directions, with electrostatic interactions computed via the particle mesh Ewald method, while short-range interactions are calculated with a truncation radius of 1.2 nm. To uphold the rigidity of the MOFs and forestall excessive structural deformation during simulation, a bias force is applied to fix the MOFs, with the force constant established at 1000 kJ mol^{-1} nm^{-1}.

3.2 Electrostatic potential (ESP) and enthalpy change (Δ*H*).

We have employed the *Gaussian 09 Revision D.011* and *Multiwfn* (version 3.8) to perform all density functional theory (DFT) calculations[8-10]. Herein, the fragments of ($Ptriaz^+$)-Anion (Anion = $^{99}TcO_4^-$ / NO_3^- / SO_4^{2-}) were chosen as the theoretical models to elaborate the relative good adsorption selectivity of $Ptriaz^+$. For ($Ptriaz^+$)-Anion complex structures, geometries were optimized at B3LYP-D3(BJ)/6-31G* or SDD level. Moreover, the electrostatic potential (ESP)

on the van der Waals (vdW) surfaces of $Ptriaz^+$ was calculated at M062X-D3/def2-TZVP level based on the optimized structure. Based on the optimized reactant and product, the calculations of single-point energies were then performed at different levels for calculating different terms of ΔH referred in Wang's work[11]. The values of ΔH were calculated by the following equations (7-9).

$$\Delta H = H_{Product} - H_{Reactant} \quad (7)$$

$$H = E_{high} + ZPE_{corr} + H_{corr} + \Delta G_{solv} + 1.89\ kcal/mol \quad (8)$$

$$\Delta G_{solv} = E_{sol} - E_{gas} + 1.89\ kcal/mol \quad (9)$$

where E_{high} is the high-precision electronic energy calculated at M062X-D3/def2-TZVP level. ZPE_{corr} and H_{corr} are the zone-point energy correction and the thermal correction to enthalpy, respectively, which obtained by the *Shermo* program[12]. G_{solv} is the solvation free energy. Here, the solvation free energy was approximated to be equal to the solvation free enthalpy. E_{sol} and E_{gas} were single point energies in the liquid phase and gas phase calculated at the M052X/6-31G* or SDD level. For E_{sol}, the SMD implicit solvent model was used. All the figures of molecular structures were plotted by VMD and CYLView software.

3.3 Molecular dynamics (MD) simulations of $^{99}TcO_4^-$ adsorption

Classical MD simulations were carried out to investigate the interactions of NO_3^-, SO_4^{2-} and $^{99}TcO_4^-$ with Ptriaz / MW-Ptriaz@MOF_C at the atomic level in the presence of an alternating electric field (electric-field-z = 0.05 V/nm). The details of the calculations were referenced from the published literature with minor modifications[13, 14]. For Ptriaz, we adopted three layers of cation polymer chains (with 12 repeat units) fixed in the center of the box. For MW-Ptriaz@MOF_C, we adopted single layer MOF_C accompanied by three cationic polymer chains threading through the nanochannels of MOFs as a representative model to investigate the competing adsorption behaviors of various anions. Furthermore, the simulation box had a size of (4.9×4.9×17.2 nm). The number of NO_3^- / SO_4^{2-} / $^{99}TcO_4^-$ anions were 50 for the upper region and 50 for the lower region. Particularly, extra 36 $TFSI^-$ / 36 NO_3^- anions were resided in the Ptriaz and MW-Ptriaz@MOF_C model, respectively. Due to $TFSI^-$ / NO_3^- anions being used as the counter anions during the synthesis of Ptriaz and MW-Ptriaz@MOF_C. The number of water molecules was 1900 for both the upper and lower regions. 200 H_3O^+ ions were added to balance the charge of the simulation system.

All MD simulations were performed with the *GROMACS* software package (version 2019.6). *VMD* software (version 1.9.3) was applied for trajectory visualization and analysis. The initial configurations were constructed using Materials Studio software, with H_2O molecules being

inserted to achieve a bulk density of 0.5 g/mL. The MOF_C was set as rigid to ensure that the atoms were fixed during the simulation. The UFF force field was employed to describe the MOFs and the GAFF force field was employed to describe the anions and Ptriaz, respectively. In the production run, energy minimization was utilized to achieve relaxation of the simulation box. Subsequently, isothermal-isochoric (NVT) ensemble was utilized with a time step of 1 fs to refine the simulation box, wherein the temperature was maintained at 300 K. Temperature control was achieved through the implementation of the Nose-Hoover thermostat. Set the cutoff distance for van der Waals and electronic interactions to 1.5 nm, and use the Particle Mesh Ewald (PME) method to calculate long-range electrostatic interactions. The optimization duration was set to 20.0 ns, ensuring the attainment of a stable system. Molecule trajectory coordinates were recorded at intervals of 10 ps steps. Throughout all MD simulations, atom motion was governed by classical Newtonian equations, resolved using the velocity-Verlet algorithm.

Supplementary Figures

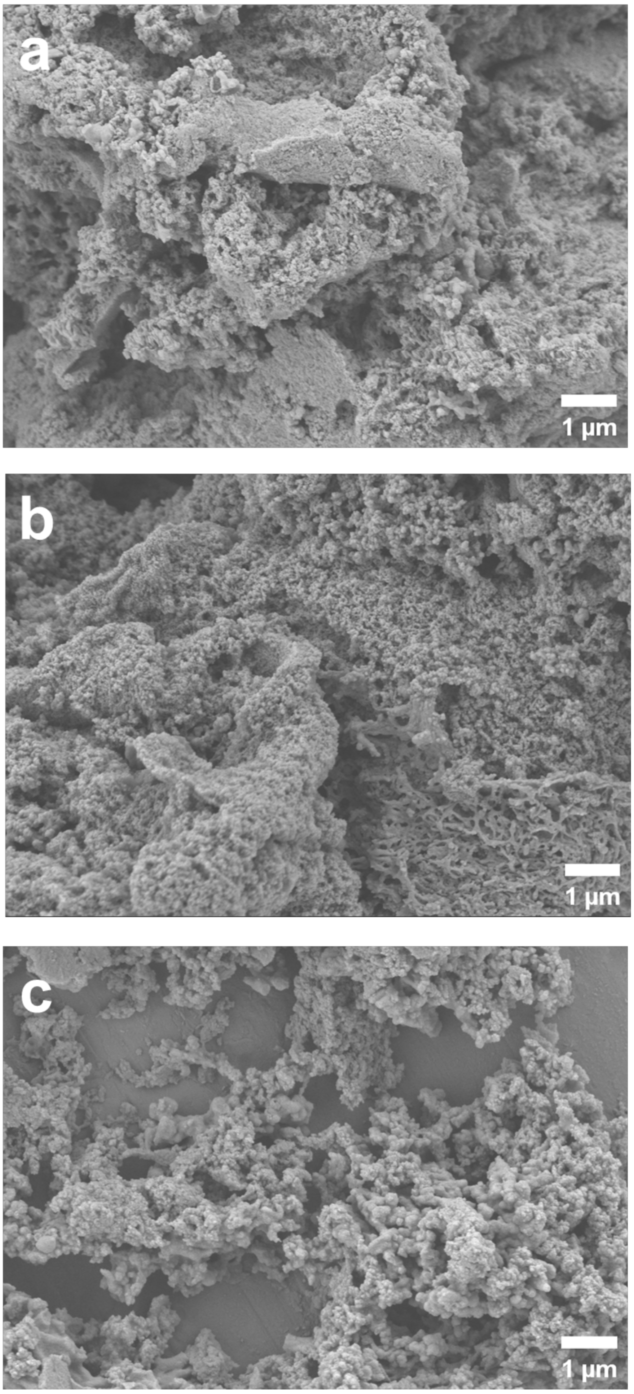

Fig. S1 (a-c) The SEM images for IPC-A, IPC-B and IPC-C.

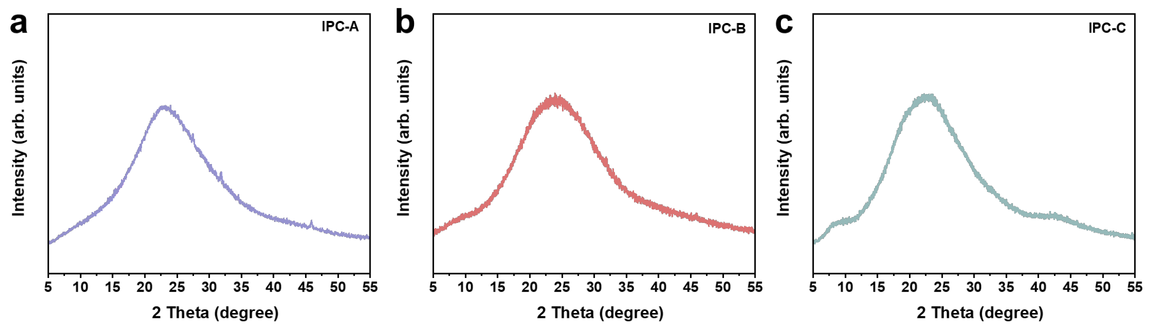

Fig. S2 (a-c) PXRD patterns for IPC-A, IPC-B and IPC-C. Source data are provided as a Source Data file.

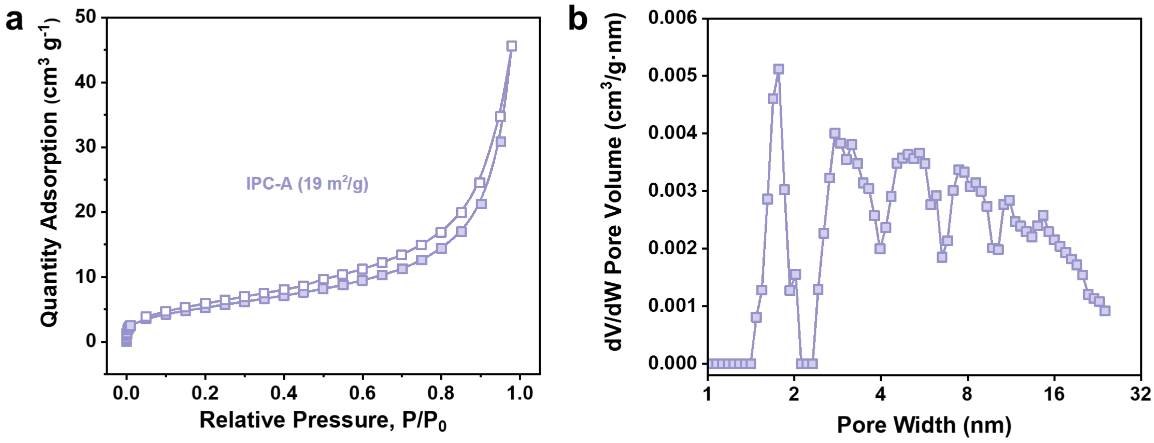

Fig. S3 (a) N_2 sorption and **(b)** pore size distribution analyses based on NLDFT method for IPC-A. Source data are provided as a Source Data file.

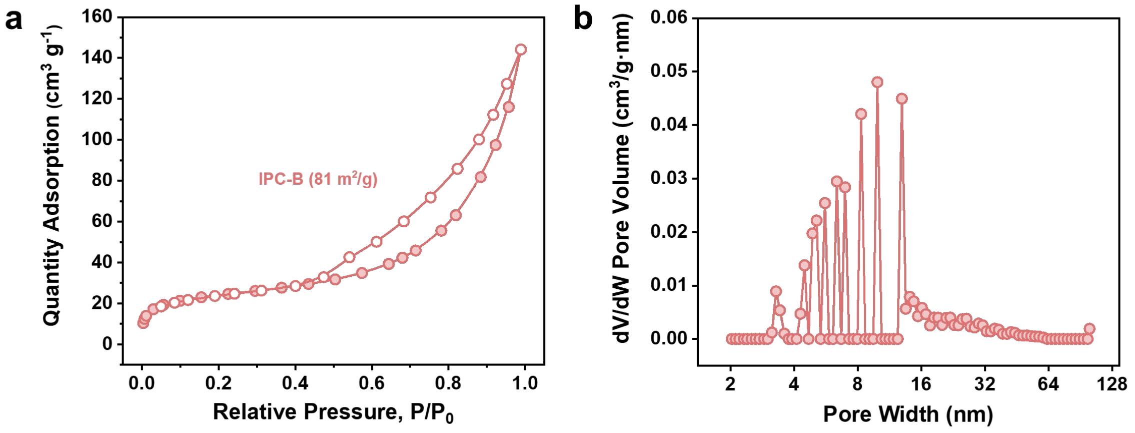

Fig. S4 (a) N_2 sorption and **(b)** pore size distribution analyses based on NLDFT method for IPC-B. Source data are provided as a Source Data file.

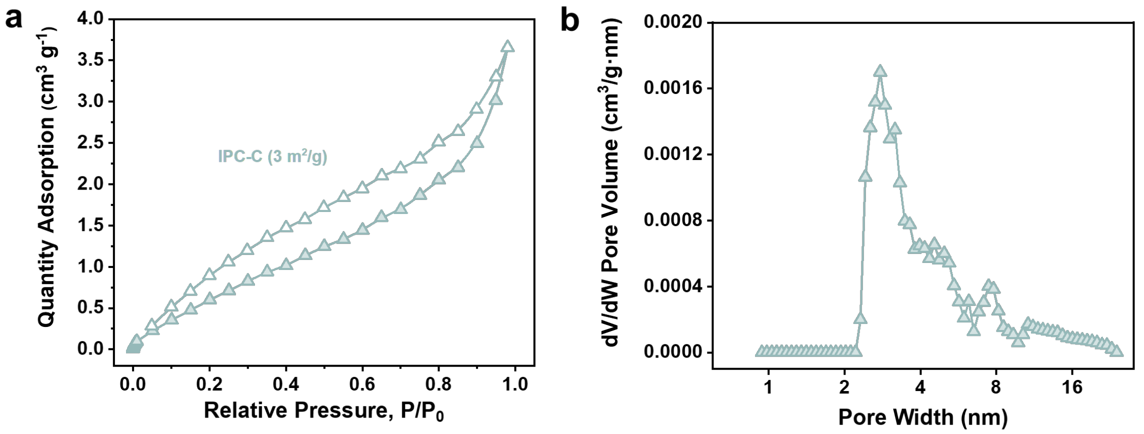

Fig. S5 (a) N_2 sorption and **(b)** pore size distribution analyses based on NLDFT method for IPC-C. Source data are provided as a Source Data file.

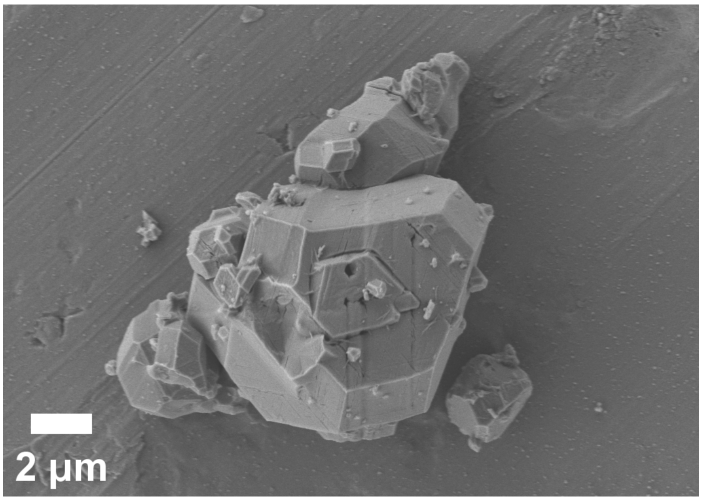

Fig. S6 The SEM image of pure MOF_A (CuBTC) particles.

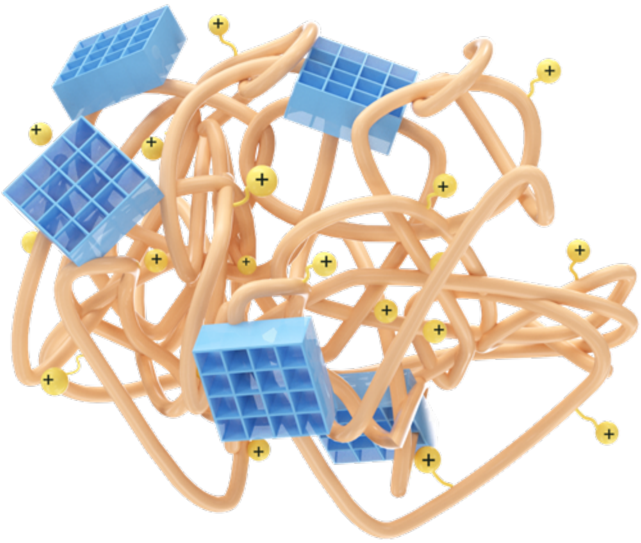

Fig. S7 Random distribution of entangled cationic polymer chains around MOFs under single acting force regulation (coordination bond).

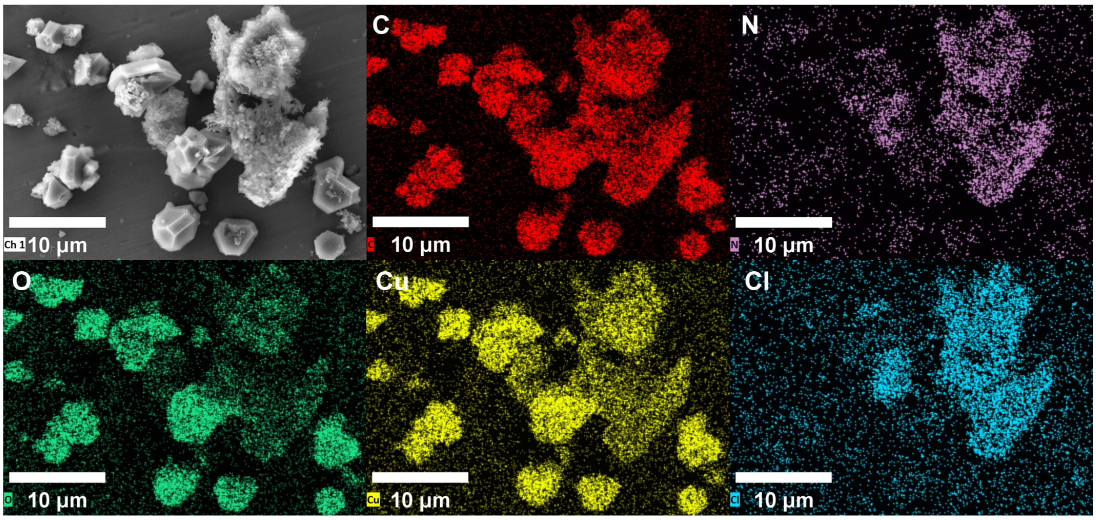

Fig. S8 The SEM-EDS mapping of NS-Ptriaz@MOF$_A$.

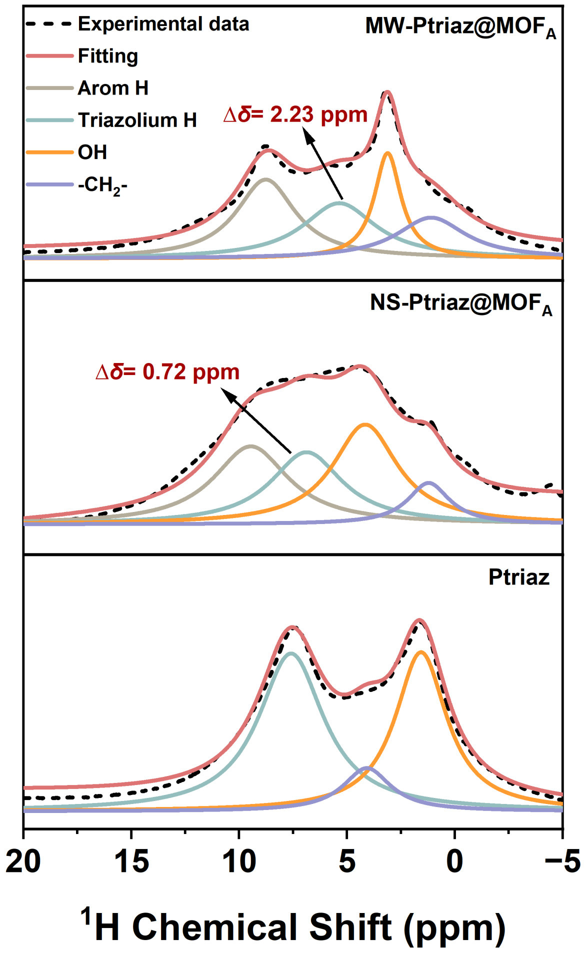

Fig. S9 High-speed magic angle spinning ^{1}H solid-state NMR of Ptriaz, NS-Ptriaz@MOF$_A$ and MW-Ptriaz@MOF$_A$, respectively. Source data are provided as a Source Data file.

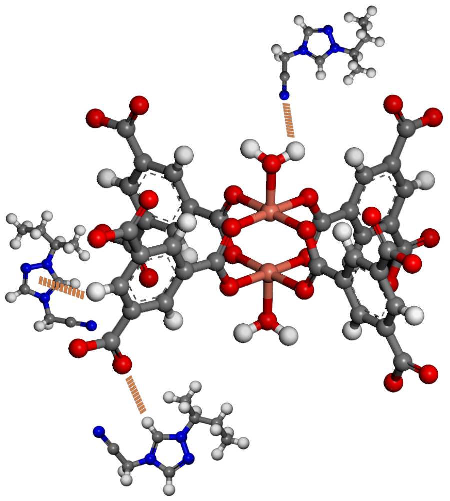

Fig. S10 Schematic for illustrating intramolecular interaction between cationic polymer chains (Ptriaz) and MOF_A (CuBTC).

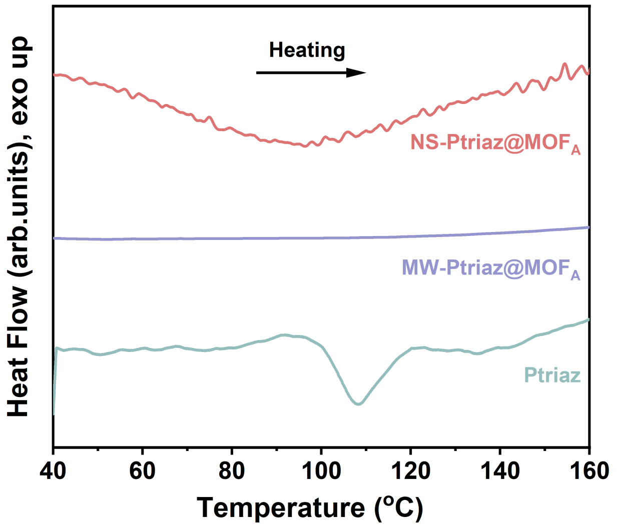

Fig. S11 Normalized DSC curves of Ptriaz, NS-Ptriaz@MOF_A and MW-Ptriaz@MOF_A, respectively. Under nitrogen atmosphere with a heating rate of 5 °C min^{-1}. Source data are provided as a Source Data file.

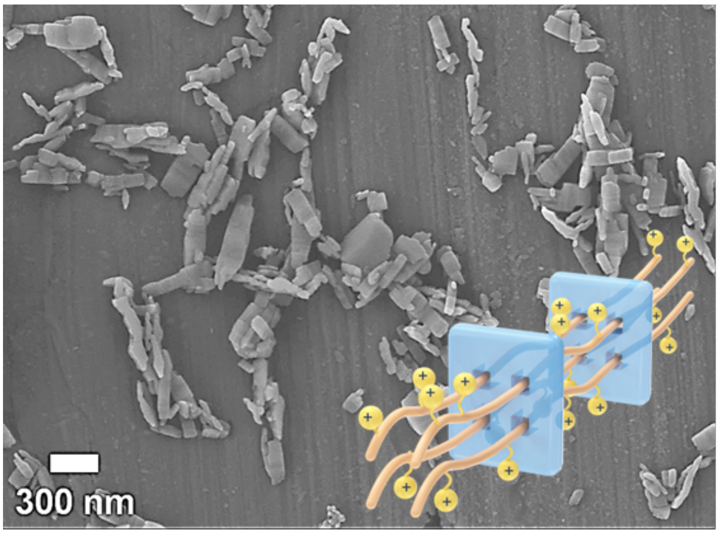

Fig. S12 A graphical representation and an SEM image of the molecularly woven polymer-MOF hybrid material synthesized using MOF_B as a template.

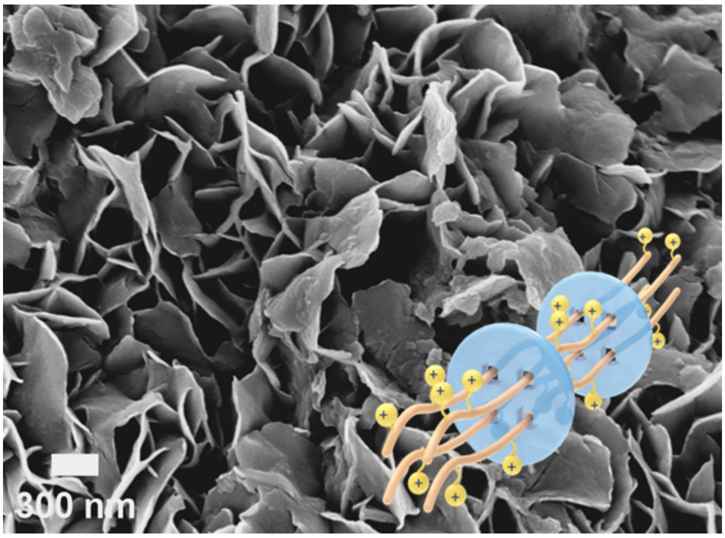

Fig. S13 A graphical representation and an SEM image of the molecularly woven polymer-MOF hybrid material synthesized using MOF_C as a template.

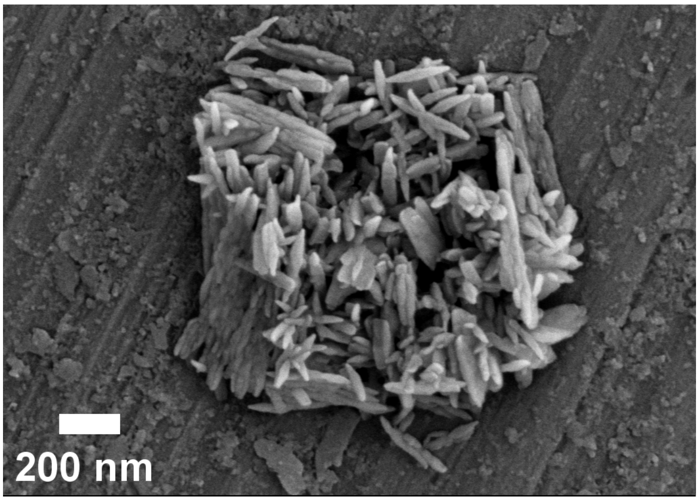

Fig. S14 The SEM image of pure MOF_B (CuBDC).

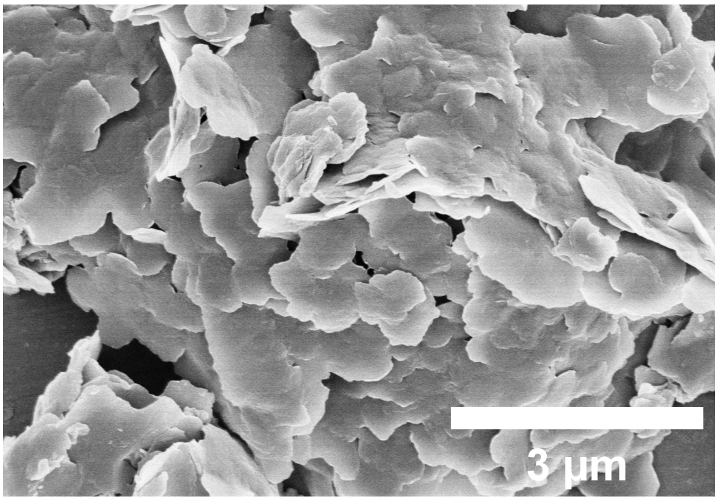

Fig. S15 The SEM image of pure MOF_C (CuTCPP).

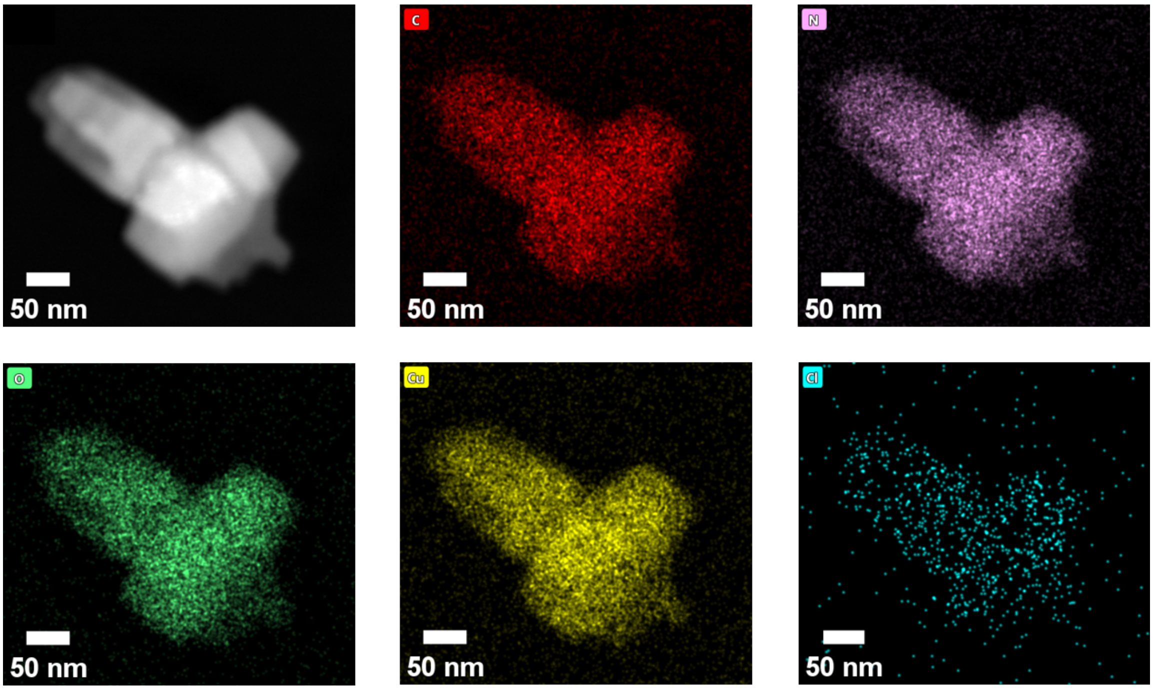

Fig. S16 STEM images and elemental mapping of MW-Ptriaz@MOF$_B$.

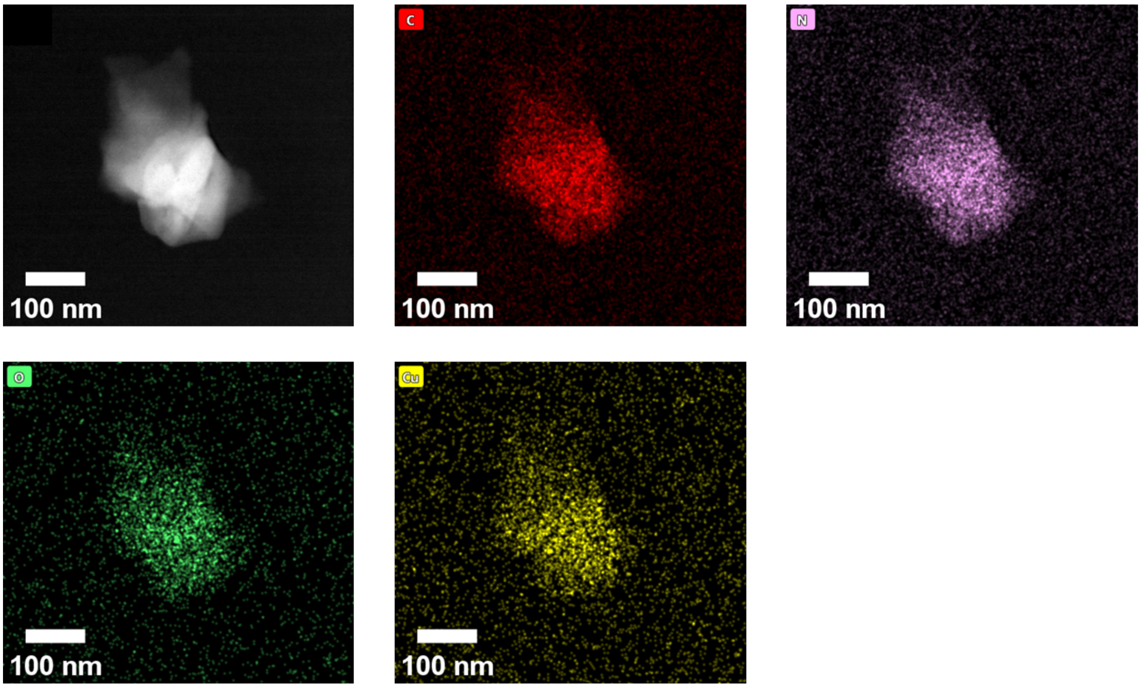

Fig. S17 STEM images and elemental mapping of MW-Ptriaz@MOF$_C$. (Since MW-Ptriaz@MOF$_C$ was synthesized using copper nitrate, it does not contain Cl elements.)

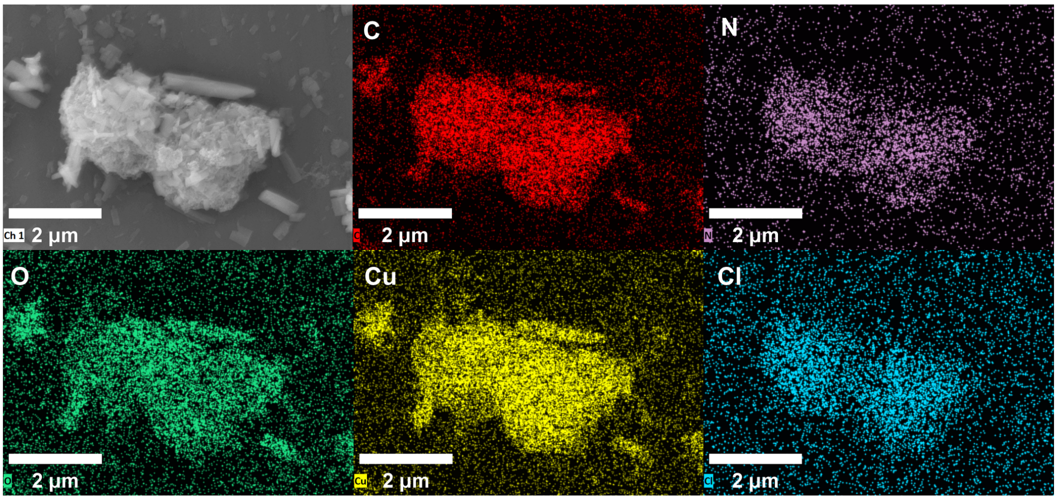

Fig. S18 The SEM-EDS mapping of NS-Ptriaz@MOF$_B$.

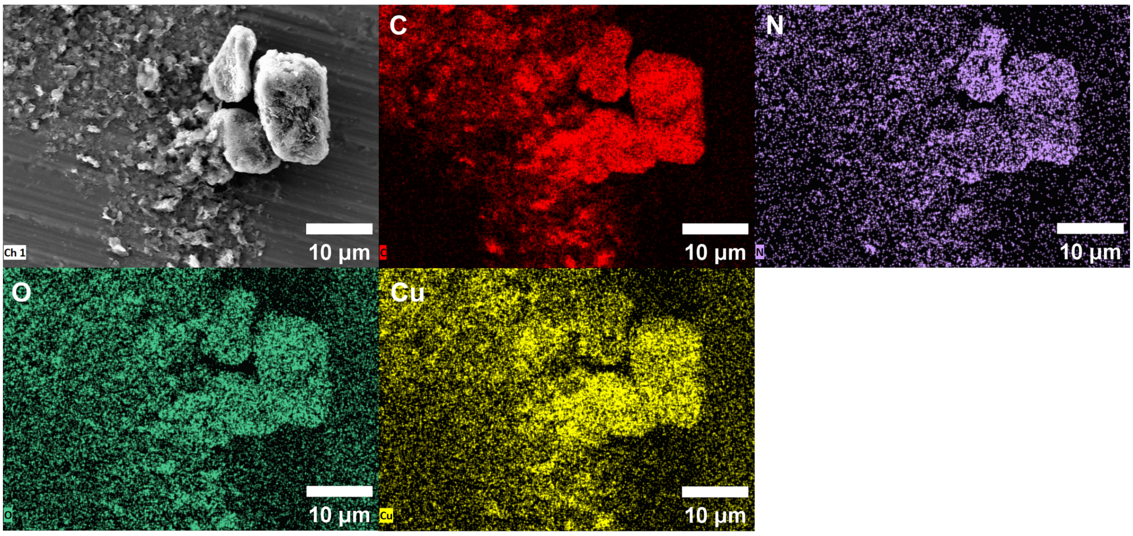

Fig. S19 The SEM-EDS mapping of NS-Ptriaz@MOF$_C$. Since NS-Ptriaz@MOF$_C$ was synthesized using copper nitrate, it does not contain Cl elements.

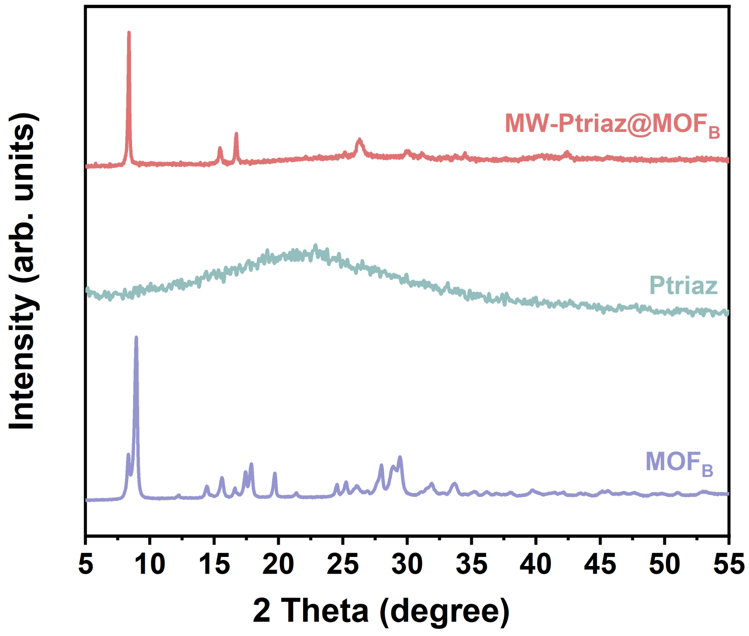

Fig. S20 PXRD patterns for MOF$_B$, Ptriaz and MW-Ptriaz@MOF$_B$, respectively. Source data are provided as a Source Data file.

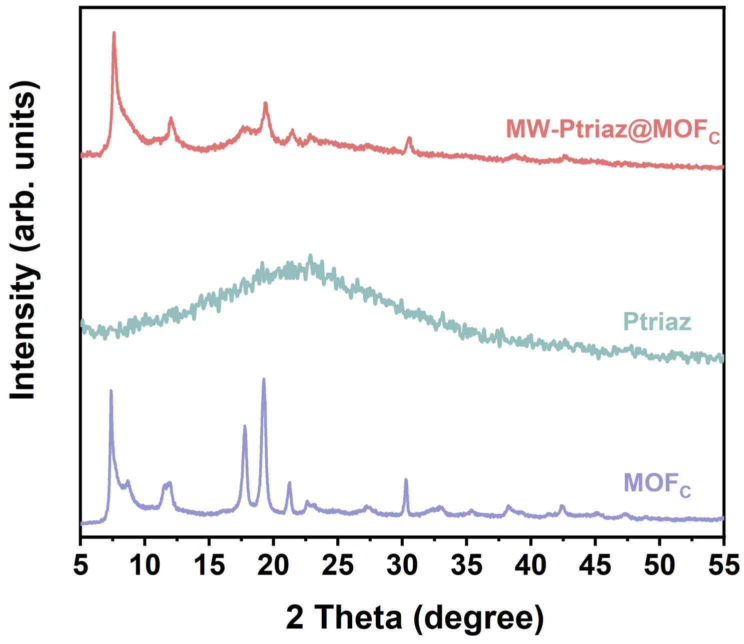

Fig. S21 PXRD patterns for MOF$_C$, Ptriaz and MW-Ptriaz@MOF$_C$, respectively. Source data are provided as a Source Data file.

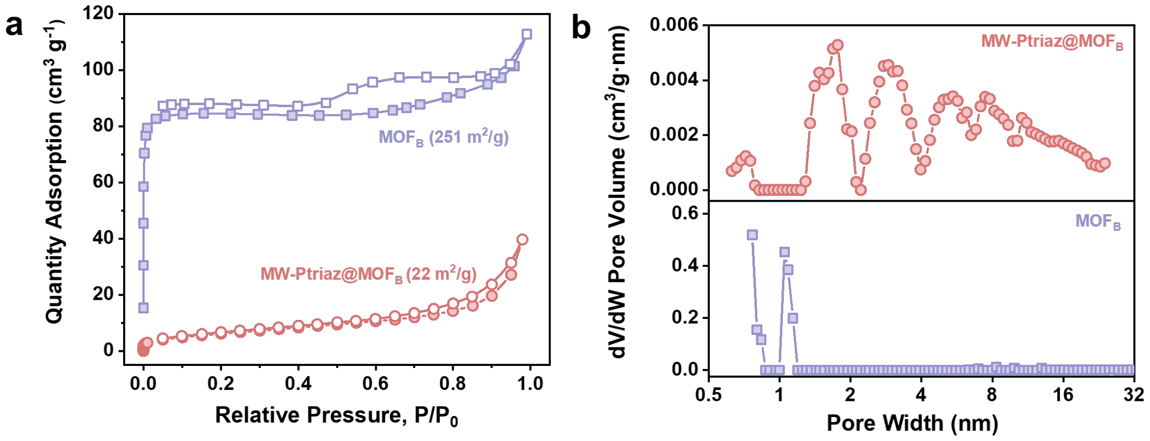

Fig. S22 **(a)** N_2 sorption and **(b)** pore size distribution analyses based on NLDFT method for MOF_B and MW-Ptriaz@MOF_B, respectively. Source data are provided as a Source Data file.

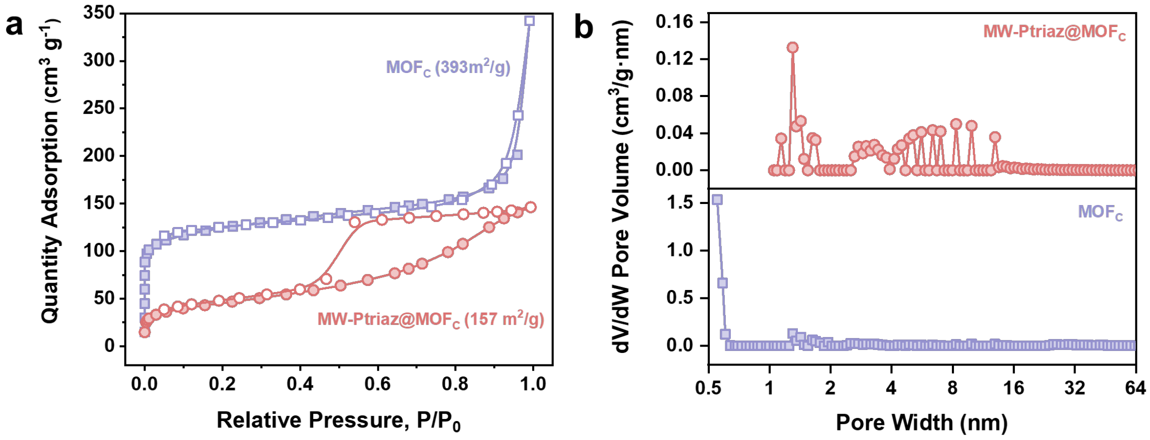

Fig. S23 **(a)** N_2 sorption and **(b)** pore size distribution analyses based on NLDFT method for MOF_C and MW-Ptriaz@MOF_C, respectively. Source data are provided as a Source Data file.

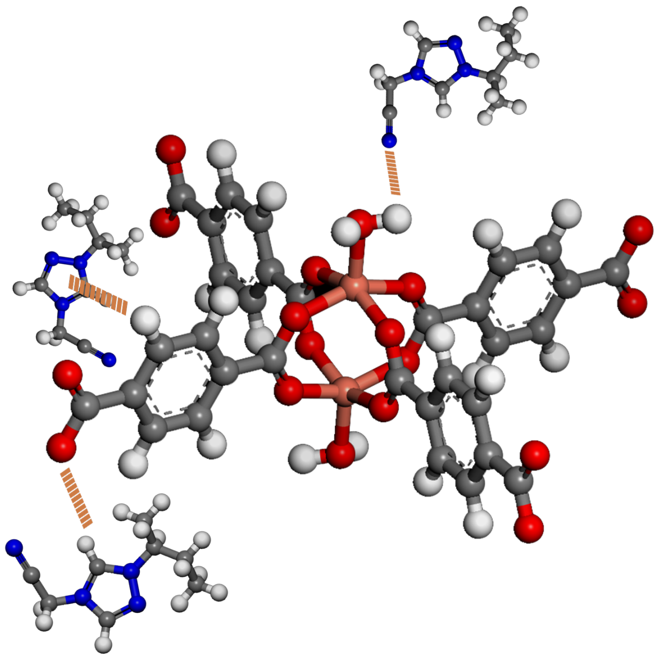

Fig. S24 Schematic for illustrating intramolecular interaction between cationic polymer chains (Ptriaz) and MOF_B (CuBDC).

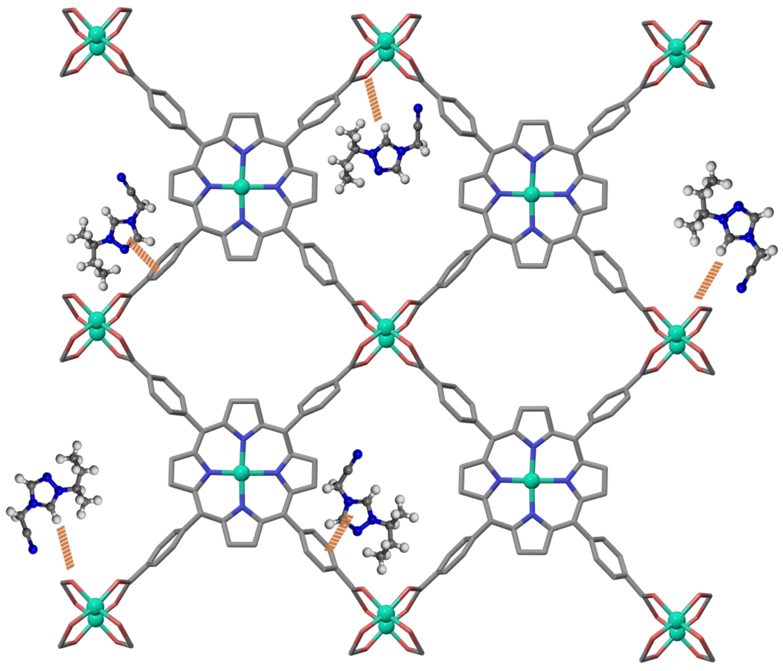

Fig. S25 Schematic for illustrating intramolecular interaction between cationic polymer chains (Ptriaz) and MOF_C (CuTCPP).

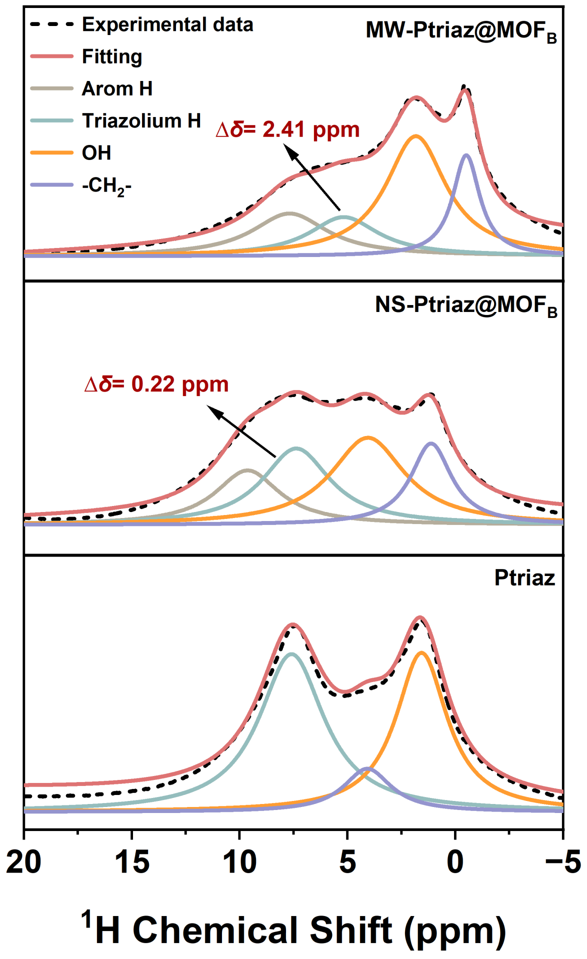

Fig. S26 High-speed magic angle spinning ^{1}H solid-state NMR of Ptriaz, NS-Ptriaz@MOF$_B$ and MW-Ptriaz@MOF$_B$, respectively. Source data are provided as a Source Data file.

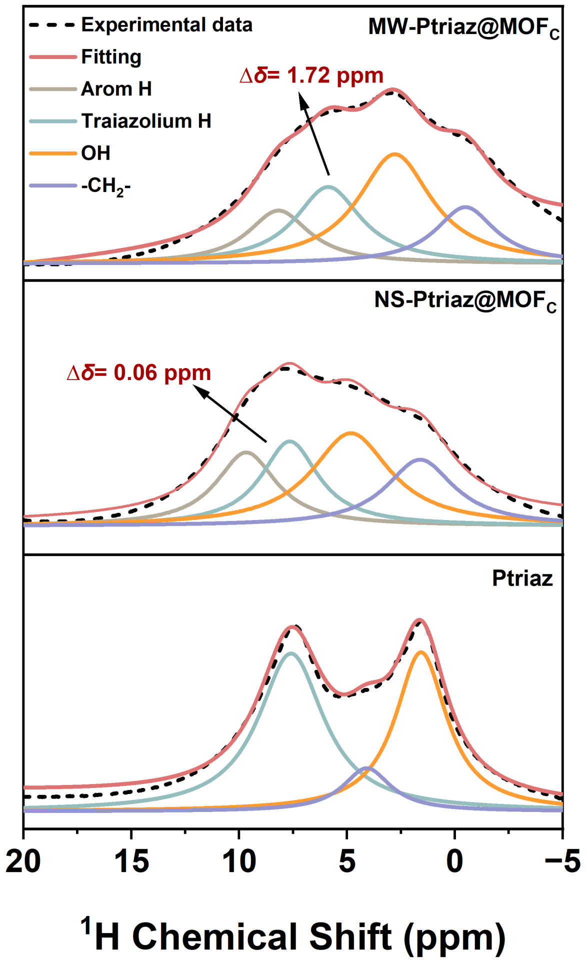

Fig. S27 High-speed magic angle spinning ^{1}H solid-state NMR of Ptriaz, NS-Ptriaz@MOFc and MW-Ptriaz@MOFc, respectively. Source data are provided as a Source Data file.

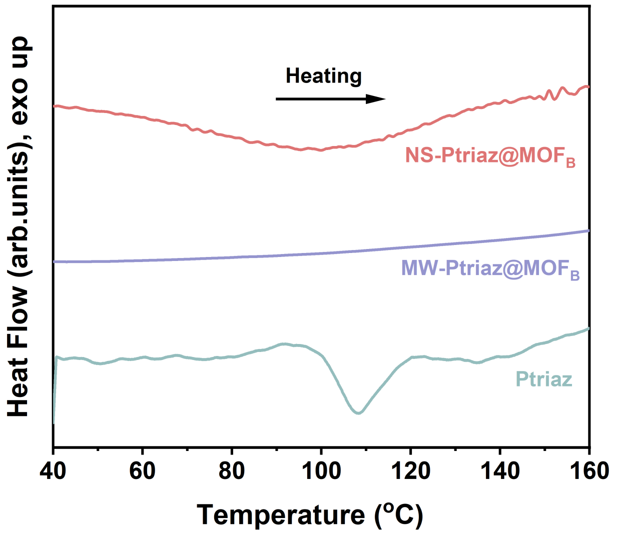

Fig. S28 Normalized DSC curves of Ptriaz, NS-Ptriaz@MOF$_B$ and MW-Ptriaz@MOF$_B$, respectively. Under nitrogen atmosphere with a heating rate of 5 °C min^{-1}. Source data are provided as a Source Data file.

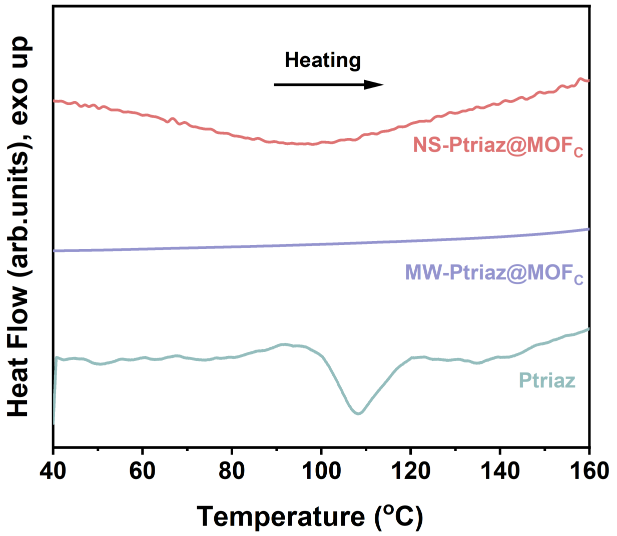

Fig. S29 Normalized DSC curves of Ptriaz, NS-Ptriaz@MOF$_C$ and MW-Ptriaz@MOF$_C$, respectively. Under nitrogen atmosphere with a heating rate of 5 °C min^{-1}. Source data are provided as a Source Data file.

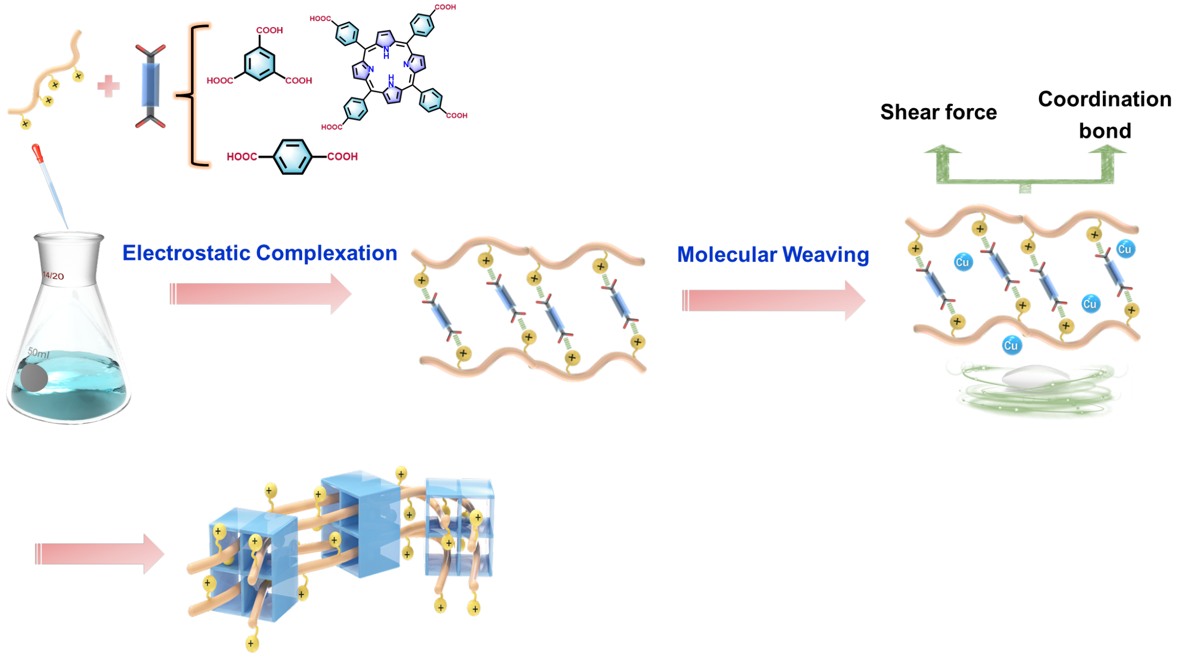

Fig. S30 Schematic illustration of synthesis strategies for ionic polymer-MOF hybrid materials via in situ molecular weaving.

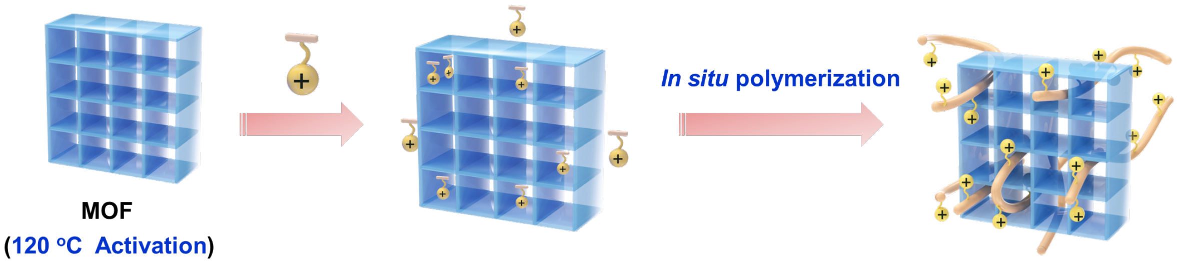

Fig. S31 Schematic illustration of synthesis strategies for ionic polymer-MOF hybrid materials via in situ polymerization.

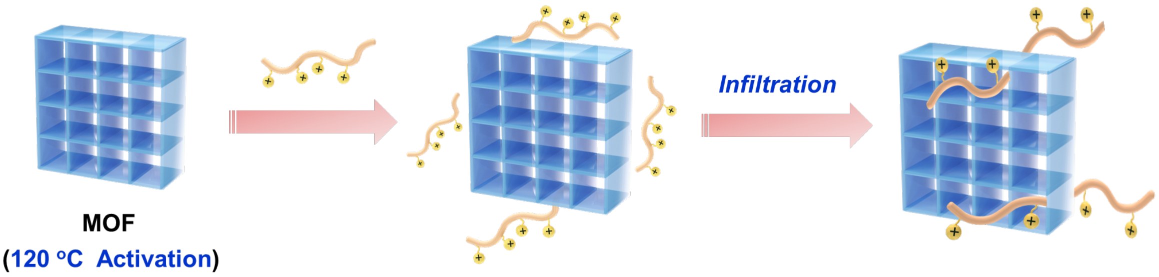

Fig. S32 Schematic illustration of synthesis strategies for ionic polymer-MOF hybrid materials via externally assisted infiltration.

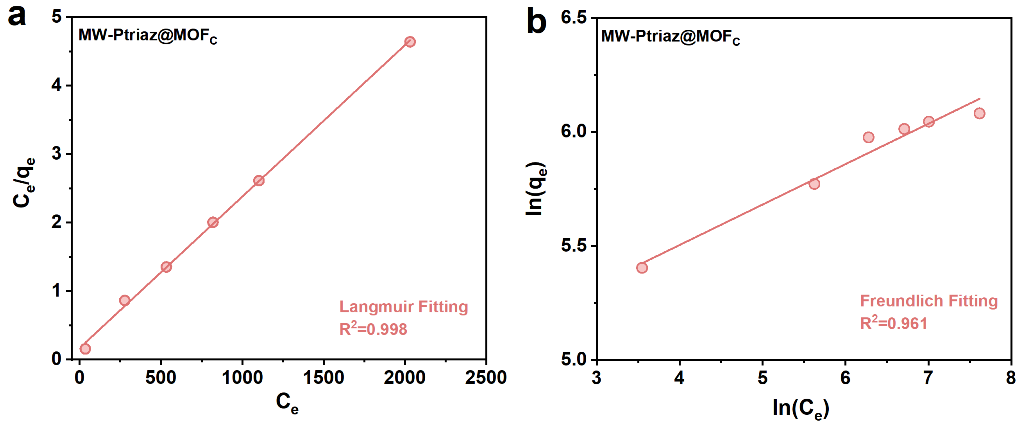

Fig. S33 Sorption isotherm of MW-Ptriaz@MOFC for ReO_4^- adsorption fitting by **(a)** Langmuir model and **(b)** Freundlich model. Source data are provided as a Source Data file.

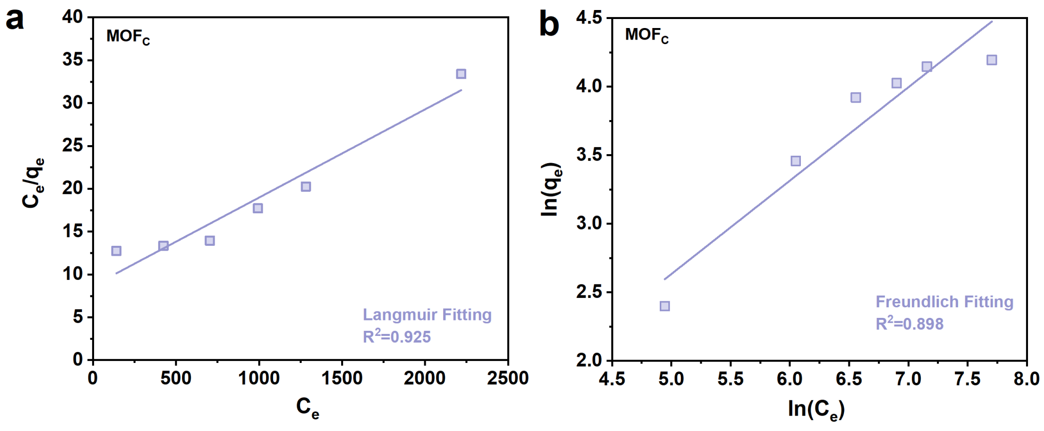

Fig. S34 Sorption isotherm of MOFC for ReO_4^- adsorption fitting by **(a)** Langmuir model and **(b)** Freundlich model. Source data are provided as a Source Data file.

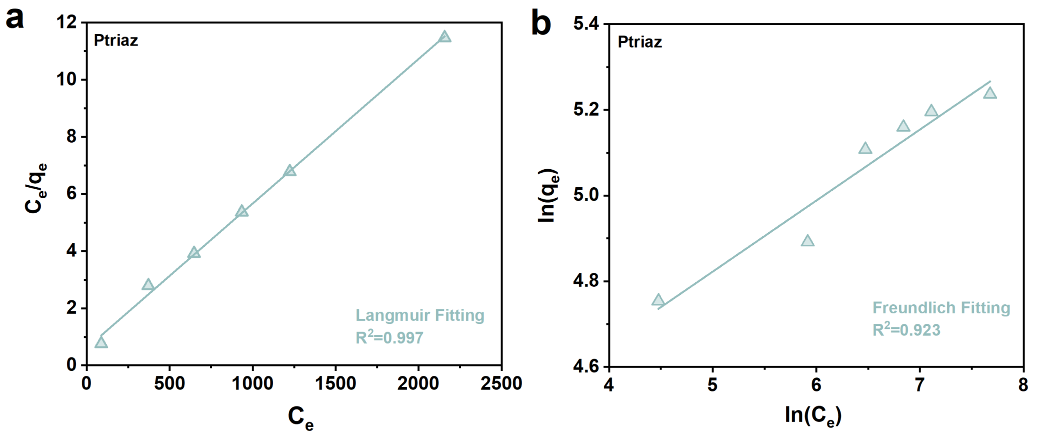

Fig. S35 Sorption isotherm of Ptriaz for ReO_4^- adsorption fitting by **(a)** Langmuir model and **(b)** Freundlich model. Source data are provided as a Source Data file.

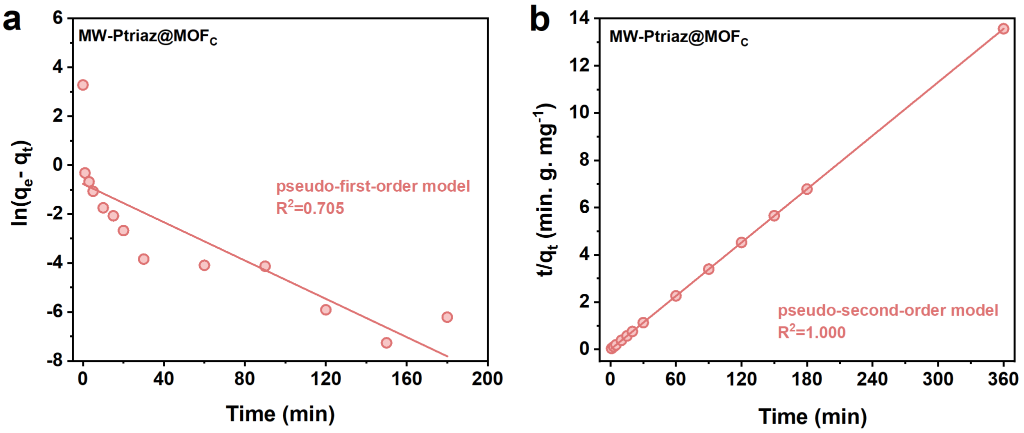

Fig. S36 **(a)** The pseudo-first-order model and **(b)** the pseudo-second-order model of MW-Ptriaz@MOF$_C$ after adsorption of ReO_4^-. Source data are provided as a Source Data file.

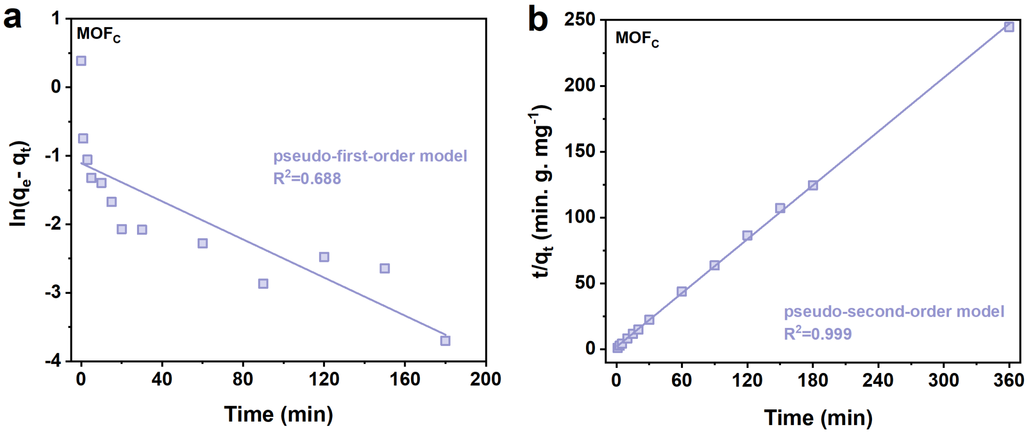

Fig. S37 **(a)** The pseudo-first-order model and **(b)** the pseudo-second-order model of MOF$_C$ after adsorption of ReO_4^-. Source data are provided as a Source Data file.

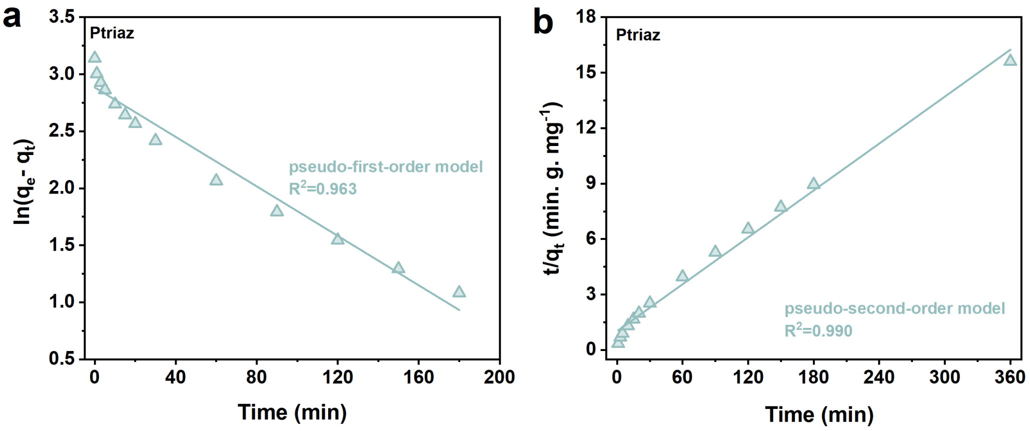

Fig. S38 **(a)** The pseudo-first-order model and **(b)** the pseudo-second-order model of Ptriaz after adsorption of ReO_4^-. Source data are provided as a Source Data file.

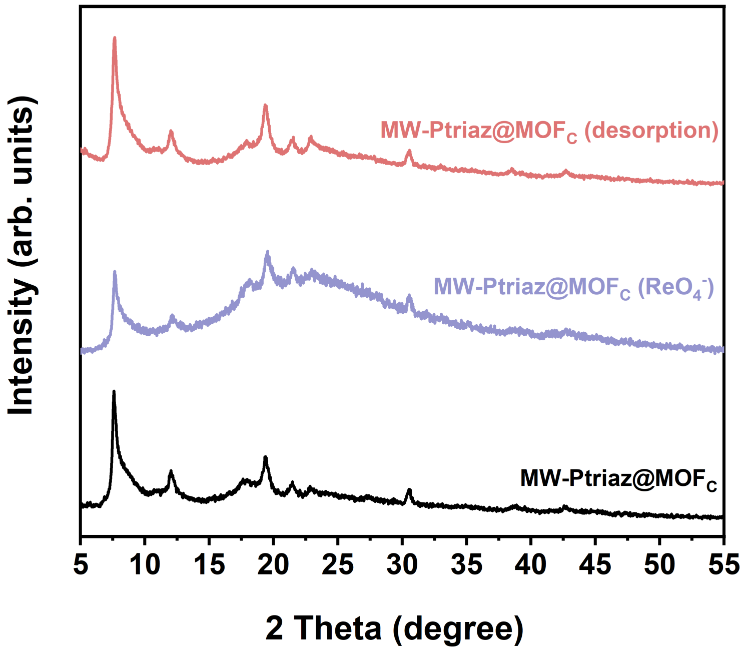

Fig. S39 PXRD patterns of MW-Ptriaz@MOF$_C$ after the ReO_4^- adsorption/desorption. Source data are provided as a Source Data file.

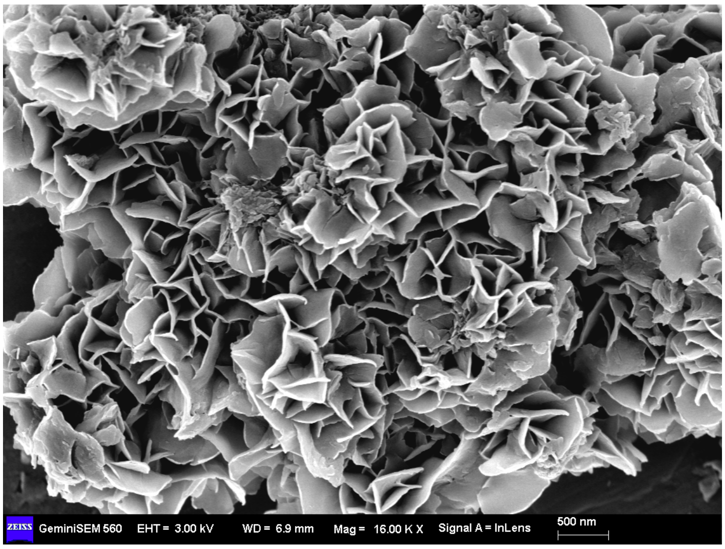

Fig. S40 SEM images of MW-Ptriaz@MOFc after regeneration.

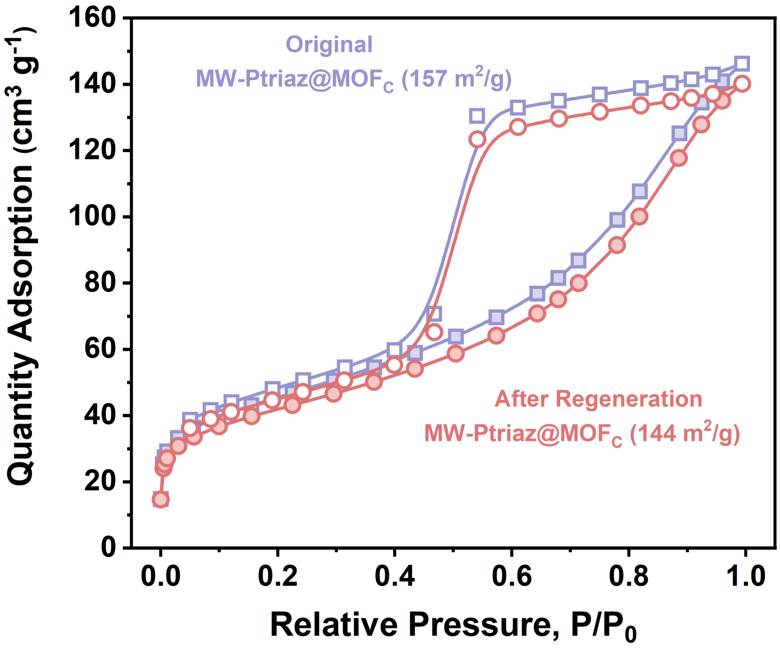

Fig. S41 N_2 sorption for MW-Ptriaz@MOFc and regenerated MW-Ptriaz@MOFc, respectively. Source data are provided as a Source Data file.

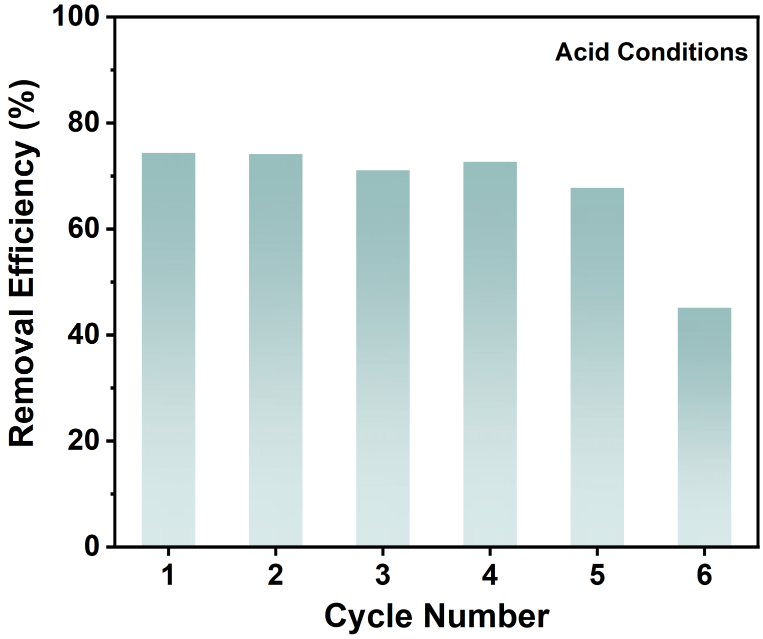

Fig. S42 Reusability of MW-Ptriaz@MOFc for ReO_4^- removal under acidic conditions (pH=1). Source data are provided as a Source Data file.

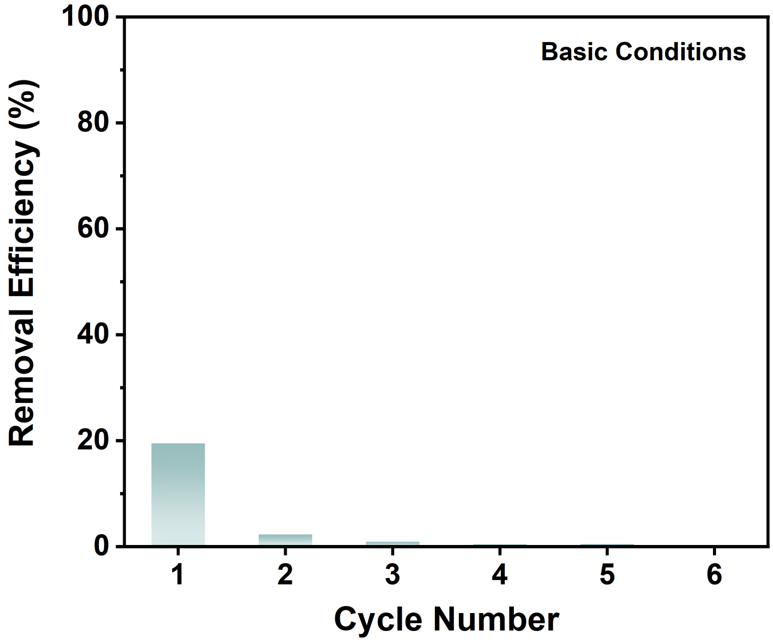

Fig. S43 Reusability of MW-Ptriaz@MOFc for ReO_4^- removal under basic conditions (pH=11). Source data are provided as a Source Data file.

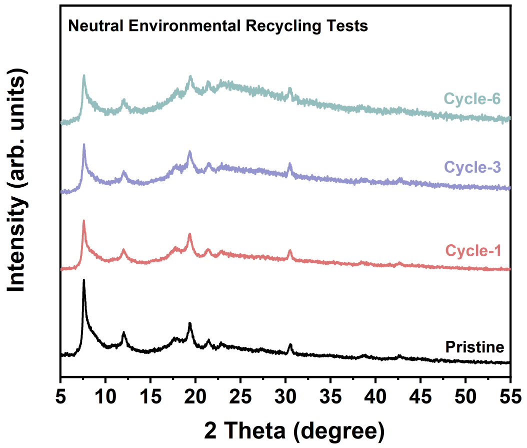

Fig. S44 PXRD patterns of MW-Ptriaz@MOFc after the recycling test in neutral environment. Source data are provided as a Source Data file.

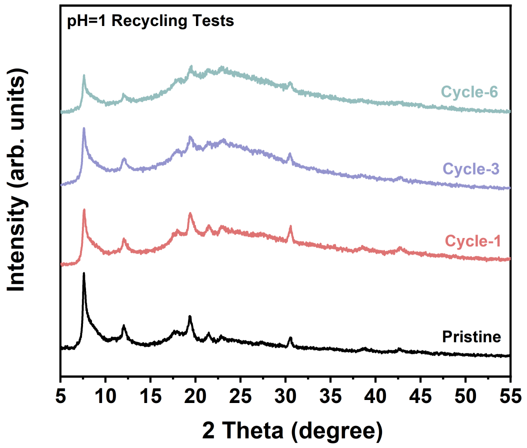

Fig. S45 PXRD patterns of MW-Ptriaz@MOFc after the recycling test in acidic environment (pH=1). Source data are provided as a Source Data file.

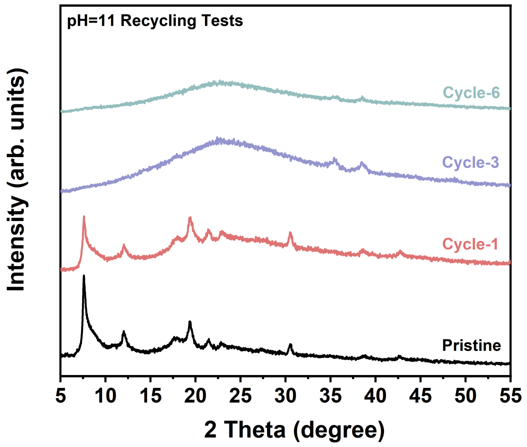

Fig. S46 PXRD patterns of MW-Ptriaz@MOFc after the recycling test in basic environment (pH=11). Source data are provided as a Source Data file.

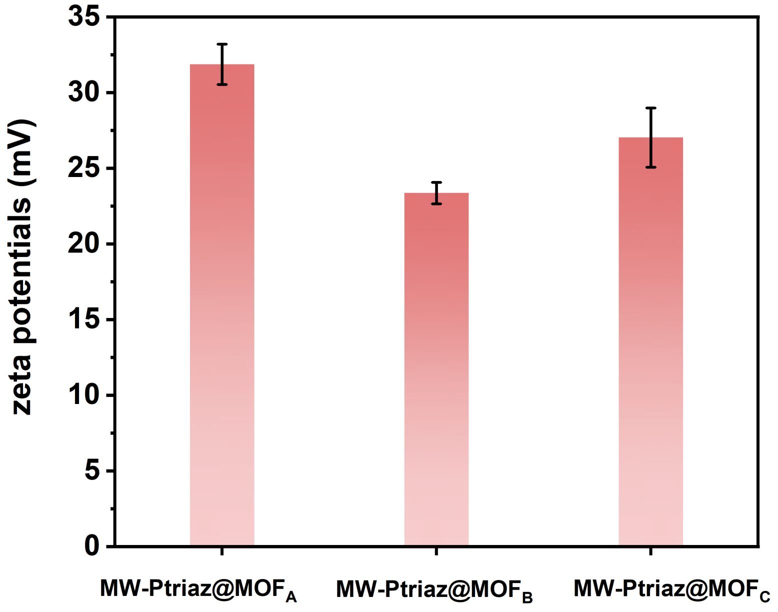

Fig. S47 The zeta potentials of molecular weaving ionic polymer-MOF hybrid materials. Error bars represent standard deviation. n = 3 independent experiments. Source data are provided as a Source Data file.

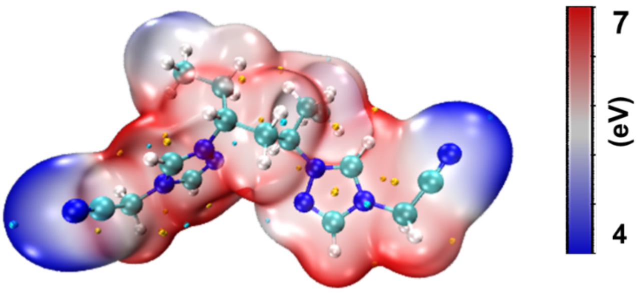

Fig. S48 Corresponding electrostatic potential distribution of $(Ptriaz^{+})_2$.

Supplementary Tables

Table S1. Comparison of three synthesis strategies

Method	Polymer loading (wt%)	Energy consumption & Processing time
Molecular weaving	A: 71.27%<br>B: 51.38%<br>C: 68.14%	Complexation: R.T., 30 mins<br>Weaving: 80°C, 4~6 h
In situ polymerization	A: 22.61%<br>B: 31.61%<br>C: 21.24%	Activation: 120°C, 12 h<br>Diffusion: R.T., 12 h<br>Polymerization: 80°C, 6 h; 110°C, 3 h
Externally assisted infiltration	A: 19.73%<br>B: 6.91%<br>C: 2.98%	Activation: 120°C, 12 h<br>Infiltration: R.T., 24 h

Table S2. Composition of simulated Hanford LAW Melter Recycle stream

Anion	Concentration, mol/L	Anion: $^{99}TcO_4^-$/ReO_4^- molar ratio
$^{99}TcO_4^-$/ReO_4^-	1.94×10^{-4}	1.0
NO_3^-	6.07×10^{-2}	314
Cl^-	6.39×10^{-2}	330
NO_2^-	1.69×10^{-1}	873
SO_4^{2-}	6.64×10^{-6}	0.0343
CO_3^{2-}	4.30×10^{-5}	0.222

Table S3. Results of ReO_4^- capture by MW-Ptriaz@MOF_C from simulated Hanford waste stream.

Simulated waste solution	Anions	Solid / liquid ratio (g/L)	Removal efficiency
Hanford waste	ReO_4^-	1:1	20.31%
	ReO_4^-	5:1	47.7%
	ReO_4^-	10:1	65.91%

References

1. Yuan, F., Yan, D., Zhang, J., Zhang, X. & Xia, T. DMF promoted embedded of melamine inside HKUST-1 for efficient Hg(II) adsorption with regenerability. *Sep. Purif. Technol.* **335**, 126211 (2024).
2. Elder, A. C., Aleksandrov, A. B., Nair, S. & Orlando, T. M. Interactions on external MOF surfaces: desorption of water and ethanol from CuBDC nanosheets. *Langmuir* **33**, 10153-10160 (2017).
3. Ma, J., Chen, G., Bai, W. & Zheng, J. Amplified electrochemical hydrogen peroxide sensing based on Cu-porphyrin metal-organic framework nanofilm and G-Quadruplex-Hemin DNAzyme. *ACS Appl. Mater. Interfaces* **12**, 58105-58112 (2020).
4. Cui, W. R. et al. Olefin-linked cationic covalent organic frameworks for efficient extraction of $ReO_4^-/^{99}TcO_4^-$. *J. Hazard. Mater.* **446**, 130603 (2023).
5. Kuhne, T. D. et al. CP2K: An electronic structure and molecular dynamics software package - Quickstep: Efficient and accurate electronic structure calculations. *J. Chem. Phys.* **152**, 194103 (2020).
6. Van Der Spoel, D. et al. GROMACS: fast, flexible, and free. *J. Comput. Chem.* **26**, 1701-1718 (2005).
7. Lindahl, E., Hess, B. & van der Spoel, D. GROMACS 3.0: a package for molecular simulation and trajectory analysis. *J. Mol. Model.* **7**, 306-317 (2001).
8. Zhang, J. & Lu, T. Efficient evaluation of electrostatic potential with computerized optimized code. *Phys. Chem. Chem. Phys.* **23**, 20323-20328 (2021).
9. Lu, T. & Chen, F. Multiwfn: a multifunctional wavefunction analyzer. *J. Comput. Chem.* **33**, 580-592 (2012).
10. Frisch, M. et al. Gaussian 09 (Revision D.01). *Gaussian 09 Revision D.01*, (2009).

11. Li, J. et al. Task-specific tailored cationic polymeric network with high base-resistance for unprecedented $^{99}TcO_4^-$ cleanup from alkaline nuclear waste. *ACS Cent. Sci.* **7**, 1441-1450 (2021).

12. Lu, T. & Chen, Q. Shermo: A general code for calculating molecular thermochemistry properties. *Comput. Theor. Chem.* **1200**, 113249 (2021).

13. Yang, X. et al. Modulating anion nanotraps via halogenation for high-efficiency $^{99}TcO_4^-/ReO_4^-$ removal under wide-ranging pH conditions. *Environ. Sci. Technol.* **57**, 10870-10881 (2023).

14. Shen, N. et al. $^{99}TcO_4^-$ removal from legacy defense nuclear waste by an alkaline-stable 2D cationic metal organic framework. *Nat. Commun.* **11**, 5571 (2020).
