# Supplementary material for: In situ molecular weaving of ionic polymers into metal-organic frameworks for radioactive anion capture
Source: Nat Commun. 2025 Aug 11;16:7393. doi: 10.1038/s41467-025-62246-3 (PMC12339677; doi:10.1038/s41467-025-62246-3)
Supplement: Supplementary file 2 — Description of Additional Supplementary Files [file 41467_2025_62246_MOESM2_ESM.pdf]

Description of Additional Supplementary Files

File Name: Supplementary Data 1

Description: The atomic coordinates of the optimized structures from related DFT calculations.

File Name: Supplementary Data 2

Description: Initial and final configurations extracted from molecular dynamics (MD) trajectories investigating the confinement-induced orientation and ordering of cationic polymer chains within MOF.

File Name: Supplementary Data 3

Description: Initial and final configurations extracted from molecular dynamics (MD) trajectories of the adsorption of various anions into Ptriaz and MW-Ptriaz@MOF_C, respectively.
